# Media Roles in Suicide Prevention: A Systematic Review

**DOI:** 10.3390/ijerph9010123

**Published:** 2012-01-04

**Authors:** Merike Sisask, Airi Värnik

**Affiliations:** 1 Central Behavior & Health Science, Estonian-Swedish Mental Health and Suicidology Institute (ERSI), 39 Õie, Tallinn 11615, Estonia; Email: airiv@online.ee; 2 Institute of Social Work, Tallinn University, 25 Narva mnt, Tallinn 10120, Estonia

**Keywords:** media, media reporting, media portrayal, suicidal behaviours, suicidality, protective effect, provocative effect, Internet, copycat effect, Werther effect, Papageno effect

## Abstract

The aim of the current systematic review was to monitor and provide an overview of the research performed about the roles of media in suicide prevention in order to find out possible effects media reporting on suicidal behaviours might have on actual suicidality (completed suicides, attempted suicides, suicidal ideation). The systematic review was performed following the principles of the PRISMA statement and includes 56 articles. Most of the studies support the idea that media reporting and suicidality are associated. However, there is a risk of reporting bias. More research is available about how irresponsible media reports can provoke suicidal behaviours (the ‘Werther effect’) and less about protective effect media can have (the ‘Papageno effect’). Strong modelling effect of media coverage on suicide is based on age and gender. Media reports are not representative of official suicide data and tend to exaggerate sensational suicides, for example dramatic and highly lethal suicide methods, which are rare in real life. Future studies have to encounter the challenges the global medium Internet will offer in terms of research methods, as it is difficult to define the circulation of news in the Internet either spatially or in time. However, online media can provide valuable innovative qualitative research material.

## 1. Introduction

In suicide prevention one of the recognised public health approaches is responsible media reporting on suicidal behaviours [1,2]. Notions about suicidal contagion after reporting cases of suicide in newspapers go back to 19th century medical literature [3]. In the literature, suicide contagion is also referred to as imitative, copycat or mass cluster suicide. Associations between media portrayal and suicidal behaviours has been a subject of research for decades. Although the media is only one feature of the social environment in which suicidal behaviours can be learned [4] and the effect is probably smaller than that of other psychosocial risk factors for suicide [5], it is a significant agent in social construction of reality, especially for vulnerable persons.

Several countries and organisations—e.g., the World Health Organisation, the Samaritans, the American Foundation for Suicide Prevention—have launched and disseminated the resources to educate and empower media professionals [6,7,8,9,10]. Studies are available, which are cross-sectional and analyse whether the method of reporting about suicidal behaviours in newspapers is in line with the recommendations of international best practices [11,12,13]. 

There is also evidence, albeit from fewer studies, that modification of reporting on suicidal behaviour is feasible and can be effective [10,14]. Several studies have measured the style of media reporting about suicide before and after recommendations for media were launched [9,15] or before and after the trainings for editors and journalists were given [16,17]. Even if the intensity of reporting about suicide can increase after these interventions, the quality in terms of preventive accent of media coverage tend to improve [17]. As these studies do not include clear outcome measure related to suicidal behaviours, the conclusions about possible suicide preventive impact of such interventions remain ambiguous. The question how does modification of media reporting influence actual suicidal behaviours is much more interesting and challenging.

The aim of the current systematic review was to monitor and give an overview of the research performed about the roles of media in suicide prevention in order to find out possible effects media reporting on suicidal behaviours might have on actual suicidality, that is on completed suicides, attempted suicides and suicidal ideation.

## 2. Experimental Section

**Figure 1:**
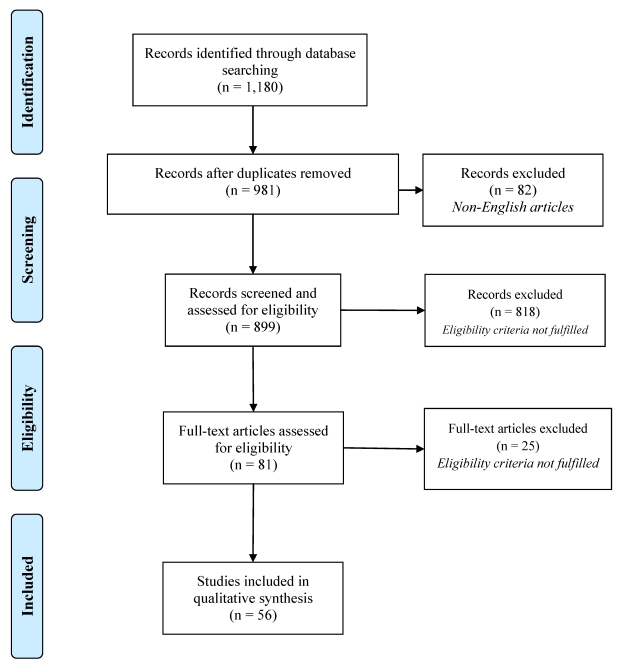
PRISMA 2009 flow diagram—Identification, screening, eligibility assessment, and inclusion of the articles.

The systematic review was performed following the principles of the PRISMA statement [18]. The results of the identification, screening, eligibility assessment, and inclusion of the articles is presented using PRISMA 2009 Flow Diagram with minor modifications (Figure 1).

A literature search of publications included in the electronic databases was conducted in July 2011 using MEDLINE (via PubMed), PsychINFO (via EBSCOHOST) and Cochrane Library. The search criteria was the occurrence of the combination of two keywords—suicid* and media—either in the title, abstracts or keywords of the publication. In total, 1,180 articles were identified through database searching (MEDLINE n = 588, PsychINFO n = 584, Cochrane Library n = 8). After the duplicates were removed, the number of publications included into the screening process was 981.

At the first stage of the screening all non-English articles were excluded (n = 82). Thereafter, all publications were screened based on the title and abstract in order to assess the relevance of the subject and eligibility. The rationale behind defining the eligibility criteria was that the influence of fictional suicide stories presented in the media has been found more controversial than the influence of non-fictional stories [19] and that purely descriptive research is unable to provide evidence about the real effect of media reporting on actual suicidal behaviours. Thus, the eligibility criteria for the inclusion were: (1) the article is a research article, a meta-analysis or a systematic review (letters, communications, descriptions of research trends and resources were excluded), (2) the research material is based on non-fictional media portrayal of suicidal behaviours, (3) the analysis of media reports has been linked with some suicidality-related outcome measure (e.g., suicide rate, non-fatal suicidal behaviours, suicidal ideation). 

In this stage of the screening 818 clearly non-relevant articles were excluded and full-texts of 81 articles were retrieved and further assessed for eligibility. After final eligibility assessment, 56 full-text articles were included in the study. Heterogeneity of the search results limited quantitative synthesis (meta-analysis) of the articles and therefore only a qualitative synthesis was performed and key findings presented.

## 3. Results and Discussion

The main characteristics and findings of the 56 studies included in qualitative synthesis are presented in Table 1. Of all studies four were meta-analysis, four were systematic reviews and 48 were research articles.

**Table 1:** Studies about the roles of media in suicide prevention: country of origin, article type, suicidality-related outcome measure and main findings.

SOURCE	COUNTRY	ARTICLE TYPE	OUTCOME MEASURE	MAIN FINDINGS
Motto 1967 [20]	USA	Research	Suicides	No significant change was revealed in suicide rates after newspaper reporting on suicidal behaviour
Motto 1970 [21]	USA	Research	Suicides	The newspaper blackout was accompanied by a significant lowering of the suicide rate in females, especially in age group 35
Phillips 1977 [22]	USA	Research	Motor vehicle fatalities	Automobile accident fatalities rose after publicized suicide stories
Phillips 1979 [23]	USA	Research	Motor vehicle fatalities	Motor vehicle fatalities (especially single-vehicle crashes) increased markedly just after publicized suicide stories
Ashton & Donnan 1981 [24]	UK	Research	Suicides by burning	Widely publicized political suicide was followed by an epidemic of copycat suicides by burning
Bollen & Phillips 1982 [25]	USA	Research	Suicides	Suicides increased shortly after a publicized suicide story
Stack 1983 [26]	USA	Research	Suicides	No relationship was found between the highly publicized mass suicide of a religious sect and national suicide rate
Wasserman 1984 [27]	USA	Research	Suicides	No significant linkage was found between the national suicide rate and stories on prominent suicides on the front page of a newspaper
Kessler *et al.* 1988 [28]	USA	Research	Suicides in teenagers	No significant association between newscasts about suicide and subsequent teenage suicides was observed
Stack 1988 [29]	USA	Research	Suicides	Publicized suicide stories during the World War I decade had no impact on suicide; peacetime suicide stories, in contrast, had significant impact
Kessler *et al*. 1989 [30]	USA	Research	Suicides	No reliable association between network news stories and suicide among adults, but significant association among teenagers existed for a specific time period
Stack 1990 [31]	USA	Research	Suicides in elderly	Months with publicized suicide stories were found to have additional elderly suicides (both male and female)
Stack 1990 [32]	USA	Research	Suicides	Stories with a victim with marital problem, such as divorce, are significantly associated with increases in suicide rates
Stack 1990 [33]	USA	Research	Suicides	Suicides of non-celebrities were associated with increase in national suicide rate, although the association was weaker than for celebrity suicide stories
Ishii 1991 [34]	Japan	Research	Suicides	Mass media has a strong increasing effect on suicides
Etzersdorfer *et al.* 1992 [35]	Austria	Research	Subway suicides and suicide attempts	After changing the quality of media reporting the number of suicides and suicide attempts in subway decreased
Stack 1992 [36]	USA	Research	Suicides	Publicized stories concerning political leaders' suicides were associated with subsequent suicides, for others there is little supporting evidence
Stack 1993 [37]	USA	Research	Suicides	Media coverage of suicide stories influences suicides independent of economic conditions
Sonneck *et al*. 1994 [38]	Austria	Research	Subway suicides and suicide attempts	Subway suicides and suicide attempts increased after dramatic media reporting, but decreased markedly after implementation of media guidelines
Hassan 1995 [39]	Australia	Research	Suicides	Suicide rates increased significantly after the publication of suicide stories in media
Jobes *et al.* 1996 [40]	USA	Research	Suicides and suicide crisis calls	After celebrity suicide the expected Werther effect did not appear, but suicide crisis calls increased significantly
Stack 1996 [41]	Japan	Research	Suicides	There is a media-suicide linkage similar in magnitude to that reported in the USA, but imitative effect is restricted to stories about Japanese suicides
Etzersdorfer & Sonneck 1998 [42]	Austria	Research	Subway suicides and suicide attempts	Number of subway suicides and suicide attempts dropped after media guidelines were developed and media campaign launched
Stack 2000 [43]	Several	Meta-Analysis	Suicides	The greater the amount of media coverage on suicide the greater the increase in suicide rate, especially if celebrity suicides and non-fictional stories were reported in newspapers
Chung & Leung 2001 [44]	Hong Kong	Research	Suicides by charcoal burning	Charcoal burning suicides became more prevalent because it was highly publicized
Etzersdorfer *et al.* 2001 [45]	Austria	Research	Suicides	The overall number of suicides increased slightly, but suicides by firearm significantly after news of celebrity suicide by gun were reported
Pirkis & Blood 2001 [46]	Several	Systematic review	Actual suicidal behaviour	There is an association between suicidal behaviour and media reporting, which satisfies sufficient of the criteria to be deemed causal
Stack 2002 [47]	Several	Meta-Analysis	Suicides	The greater the amount of media coverage on suicide the greater the increase in suicide rate, especially if celebrity suicides and non-fictional stories were reported in newspapers
Stack 2003 [48]	Several	Meta-Analysis	Suicides	The greater the amount of media coverage on suicide the greater the increase in suicide rate, especially if celebrity suicides and non-fictional stories were reported in newspapers
Etzersdorfer *et al.* 2004 [49]	Austria	Research	Suicides by firearm	The number of suicides by firearm increased after the reporting of celebrity suicide by gun
Hittner 2005 [50]	USA	Research	Suicides	A re-analysis of two classic research articles on media influence provided only partial support for the Werther effect
Mann *et al.* 2005 [1]	Several	Systematic review	Suicides	Media blackouts on reporting suicide have coincided with decrease in suicide rates, but no published studies have evaluated the impact of establishing media guidelines
Reisch & Michel 2005 [51]	Switzerland	Research	Suicides by jumping	The data suggest a regional increased popularity of the suicide method (jumping) in the period of high media attention
Shoval *et al.* 2005 [52]	Israel	Research	Suicides	Reported televising of a promo for a documentary on suicide may raise the risk of suicide in vulnerable population, especially the use of particular method (jumping)
Stack 2005 [53]	Several	Meta-Analysis	Suicides	Copycat effect was more likely reported for celebrity suicides and female suicides and less likely if studies were based on television stories and stressed negative definitions of suicide
Sudak & Sudak 2005 [54]	Several	Systematic review	Suicides	The number of suicides increased, if the media romanticized and dramatized the description of suicidal deaths
Tousignant *et al.* 2005 [55]	Canada	Research	Suicides	A celebrity's suicide was instrumental for a number of suicide in the period immediately following the event, although the size of the effect remains unknown
Pirkis *et al.* 2006 [56]	Australia	Research	Suicides	There may be an association between the quantity of media items and the number of subsequent suicides
Romer *et al.* 2006 [57]	USA	Research	Suicides	The results confirm the effect of media-induced suicide contagion
Yip *et al.* 2006 [58]	Hong Kong	Research	Suicides	There was a significant increase in suicides following the celebrity death, particularly in a subgroup of males aged 25-39 years, many of whom died by jumping
Cheng *et al.* 2007 [59]	Taiwan	Research	Suicidal behaviours (thoughts, attempts)	Strong association was found between inappropriate media reporting of celebrity suicide and subsequent suicidal behaviour (thoughts attempts) in depressed patients
Cheng *et al.* 2007 [60]	Taiwan	Research	Suicide attempts	Number of suicide attempts increased markedly and identification was self-reported after media reporting began
Cheng *et al.* 2007 [61]	Taiwan	Research	Suicides	Number of suicides increased markedly and strong modelling effect (sex, method) occurred after media reporting
Fu & Yip 2007 [62]	Hong Kong	Research	Suicidal ideation	Celebrity suicide had long-term effect on suicidal ideation (suicidal thoughts in community), both in vulnerable and non-vulnerable persons
Hagihara *et al.* 2007 [63]	Japan	Research	Suicides	Newspaper articles about suicide were a predictor of suicide for both male and female subjects
Niederkrotenthaler & Sonneck 2007 [10]	Austria	Research	Suicides and subway suicides	The media guidelines had an impact on the quality of media reporting and on suicidal behaviour (both overall suicides and subway suicides)
Fu *et al*. 2009 [64]	Hong Kong	Research	Suicidal ideation	Individual level self-reported data showed positive association between media influences (stimulus) and suicidal ideation (response)
Fu & Yip 2009 [65]	Asian regions	Meta-Analysis	Suicides	Risk of suicide was elevated after extensive media coverage of celebrity suicides
Huh *et al*. 2009 [66]	Korea	Research	Suicides	Reporting of unusual accidental deaths and specific suicide methods (charcoal burning) may lead younger people to imitative suicidal acts
Niederkrotenthaler *et al.* 2009 [67]	Austria	Research	Suicides	Copycat effects was associated with social status (celebrity) of the reported suicides and reporting characteristics were associated with a post-report increase in similar suicides
Chen *et al*. 2010 [68]	Taiwan	Research	Suicides	Significant increase in suicides (especially among individuals of the same gender and similar age) following extensive media reporting of a celebrity suicide by charcoal burning
Chen *et al*. 2010 [69]	Taiwan	Research	Suicide attempts	Major self-reported identification occurred in respondents who attempted suicide by using the same method as a celebrity (charcoal burning)
Chen *et al*. 2011 [70]	Taiwan	Research	Suicides	Increase in the intensity of suicide news reporting increased the actual number of suicides
Kunrath *et al*. 2010 [71]	Germany	Research	Railway suicides	Number of railway suicides increased significantly after non-fictional media coverage of a fatal railway accident
Niederkrotenthaler *et al*. 2010 [72]	Austria	Research	Suicides	Coverage on positive coping in adverse circumstances as covered in media items about suicidal ideation may have protective effect and decrease suicide rates
Queinec *et al*. 2011 [73]	France	Research	Suicides	Some celebrity suicides stories were followed by increase in suicides, some were not

Most of the studies (n = 19) came from North-America (the USA and one from Canada), the studies from the USA were especially over-represented before 1990. The next big region of origin of the studies was Asia (n = 16), followed by Europe (n = 12). Two studies were performed in Australia. Meta-analysis and systematic reviews had no specifically defined region or included studies from several regions.

The vast majority of the studies support the idea that media coverage of suicidal behaviours and actual suicidality are associated. Only four studies found no significant associations [20,26,27,28] and five studies expressed hesitations about clear associations or reported incoherent results [29,30,36,50,73]. All studies reporting no associations were conducted before 1990. Unfortunately it is impossible to evaluate whether these one-way results, *i.e.*, either positive or negative significant associations between media reporting and suicidality, reflect the real situation. There is a risk of reporting bias in the sense that researchers are eager to report meaningful positive results, but could keep silent if the results are not beneficial.

Mass media imitation theory presumes that if modelling works in one way (copycat suicides), it can work also on the other way (positive model) [74]. For indicating negative, provoking effect of media portrayal, the expression ‘Werther effect’ was introduced by Phillips [75] already decades ago and for the opposite, preventive effect the expression ‘Papageno effect’ was proposed recently by Niederkrotenhaler and colleagues [72]. The ‘Werther effect refers to Goethe’s novel *The sorrows of Young Werther (1774)*, where a young man takes his life for love by shooting himself. The ‘Papageno effect’ refers to Mozart’s opera *The Magic Flute (1791)*, where a young man in love becomes suicidal, but copes well thanks to his friends’ intervention. Based on the results of the current systematic review more research is available about provocative outcomes of irresponsible media reports inducing an increase in suicidal behaviours after publicized suicide stories than for protective effects. Only six research articles evaluated the protective effect of media coverage, caused either by newspaper blackout, by reducing the quantity of reporting or by changing the quality of media reporting, and observed subsequent decrease in suicidality [10,21,35,38,42,72]. 

It has been argued that certain individuals are more vulnerable to incorporate the idea and act of suicide into their concepts of self and one possible source for learning is media [76]. Several studies included in the current review proposed the suicide contagion effect of media reporting to be present only for specific vulnerable groups, as the degree of media influence is contingent on audience receptivity [36]. Subsequent similar suicides indicate strong modelling effect based on age [21,30,31,58,66,67,68] and gender [21,53,58,61,67,68]. These notions can be explained by differential identification theory, which takes age and gender as key dimensions of social life upon people build a sense of identity [77]. The fact that media-induced contagious effect has been highlighted more among young people and especially among elderly leads to the conclusion that suicide rates of the middle-aged people are less related to imitative suicide [74,77]. This is probably due to relatively low level of suicidogenic life conditions for middle-aged people. Unlike elderly, the middle-aged people have comparatively good health and are financially well off. Compared to the young, they are more settled into society’s institutional framework, including marriage, the family, work and politics [77].

One important issue which can not be neglected when assessing media influence is reporting on specific suicide methods. Studies included in the current systematic review revealed how media reports ‘advertised’ several dramatic and highly lethal suicide methods: burning [44], charcoal burning [44,66,68,69], shooting [49], jumping [51,52,58], railway suicide [71], and subway suicide [35,38,42]. Studies about newsworthiness of suicide have found that media reports are not representative to official suicide data and tend to exaggerate certain types of suicides, like suicides by celebrities and suicides involving unusual circumstances or methods [78]. The methods of drowning, jumping, shooting and rare methods are more likely to be reported than hanging [79], which is actually the most frequent suicide method in many countries.

Most of the studies have focused on short-term effect (e.g., from 1–2 days to 3–4 weeks) of media reporting, which is highly relevant period for provoking fatal and non-fatal suicidal acts. However, suicidal ideation (suicidal thoughts in the community) have found to be influenced by a publicized celebrity suicide for a longer, approximately 1-year period [62]. The long-term effect becomes probably even more important in the contemporary world, where majority of the newspapers are electronic and easily accessible in the Internet for an undetermined period of time. The Internet is global medium, which makes it difficult to determine specific area and exact dose of media reporting. These facts challenge research methods for evaluating associations between media reporting and subsequent suicidal behaviours.

The majority of studies about associations between media and suicidality follow the tradition of quantitative evaluation of media reporting, which fails to take into account the capacity of audience to make meaning out of message. Quantitative method could be complemented by examining qualitatively the multiple meanings that audiences give to media messages [80]. Niederkrotenthaler and colleagues [72] have applied both content analysis and quantitative evaluation and reached to novel findings about possibilities to provide suicide-protective effect by advertising positive coping in adverse circumstances. Even more, the readers can be active agents in constructing reality in media. For example, online newspapers provide possibilities for interaction between the suicide story and the reader, but also between the readers themselves, which in turn can construct the reality of exposed persons. Some hints about social construction and interpretation of suicide stories presented in online media can be obtained by exploring the content of spontaneous readers’ comments on media portrayal of a suicide story [13,81].

## 4. Conclusions

The vast majority of the studies about possible effects media reporting on suicidal behaviours might have on actual suicidality (fatal and non-fatal suicidal acts or suicidal ideation) support the idea that these two phenomena are associated. However, there is a risk of reporting bias in the sense that only positive results could be considered as worth to publish and zero results may remain unknown. This is especially true for the studies performed during the last two decades and investigating merely a single suicide story. Only four studies [20,26,27,28] included in the current systematic review found no significant associations and all of them were conducted before 1990.

More research is available about how irresponsible media reports can provoke suicidal behaviours (the ‘Werther effect’) and less about protective effect media can have by newspaper blackout or by changing the quality and content of media reporting (the ‘Papageno effect’). Contemporary tendencies in public health encourage researchers to ascertain protective factors as opposed to risk factors, although most of the time these are just two sides of the same coin.

Strong modelling effect of media coverage on suicide is based on age and gender. Individuals with demographic background similar to the person who committed highly publicized suicidal act (in most of the cases celebrities) are more vulnerable and receptive for identification. As it is true for suicide prevention as a whole, the research on media effects should also be target group specific, because universal approaches are less promising.

Several studies have revealed how media reports tend to ‘advertise’ dramatic and highly lethal suicide methods (burning, charcoal burning, shooting, jumping, railway and subway suicides), which are rare in real life. Media reports are not representative to official suicide data and tend to exaggerate sensational suicides.

The studies about media reporting on suicide come from a limited number of countries, although from different regions of the world. Researcher should be encouraged to assess the situation in different countries, although the fact about globalisation of news via Internet should kept in mind. Future studies have to encounter the challenges the global medium Internet will offer in terms of research methods. Circulation of news in the Internet is not limited either spatially or in time. However, online media can provide valuable innovative qualitative research material like readers’ spontaneous comments on media portrayals, which enables to analyse different meanings audience gives to messages and also interactions between different agents in this process.

One possible limitation of the study is that if alternative search regimens were utilized, additional studies might have been found. Especially the studies from earlier periods (e.g., from 1960’s to 1980’s) are difficult to find using standard search engines, an example of such omission could be the study performed by Stack [82]. The bibliographies of articles and books need to be searched, which is very time consuming. After all, it is difficult to get copies of many of these papers that are simply not accessible online. However, the authors believe that the conclusions may be largely the same, if the search procedure was amended, so this may not be as serious limitation as one may think.

One of the inclusion criteria for the current systematic review was that the study should be based on non-fictional media portrayal of suicidal behaviours, as the influence of fictional suicide stories presented in the media has been found more controversial [19]. However, the research on the description of portrayals of suicide not just in the news, but also in the movies might be of great interest. To the extent that portrayals of suicide in the cinema are inaccurate, they can contribute to public misunderstandings of the nature of suicide and hurt the development of effective suicide prevention programs. Given that people spend more time watching movies than any other leisure time pursuit, it is important to develop and apply suicide guidelines for film. Suicides in film are generally insistent with media guidelines and, for example, give graphic details on suicide methods, depict the actual, often bloody, suicide act in progress, and de-emphasize mental illness risk factors in suicides [83].

## References

[1] Mann J.J., Apter A., Bertolote J., Beautrais A., Currier D., Haas A., Hegerl U., Lonnqvist J., Malone K., Marusic A. (2005). Suicide prevention strategies: A systematic review. JAMA.

[2] Wasserman D., Wasserman D. (2001). Strategy in Suicide Prevention. Suicide: An Unnecessary Death.

[3] Leonard E.C. (2001). Confidential death to prevent suicidal contagion: An accepted, but never implemented, nineteenth-century idea. Suicide Life Threat. Behav..

[4] Schmidtke A., Häfner H., Diekstra R.F.W., Maris R., Platt S., Schmidtke A., Sonneck G. (1989). Public Attitudes towards and Effects of the Mass Media on Suicidal and Deliberate Self-Harm Behavior. Suicide and Its Prevention: The Role of Attitude and Imitation.

[5] Velting D.M., Gould M.S., Maris R.W., Silverman M.M., Canetto S.S. (1997). Suicide Contagion. Review of Suicidology, 1997.

[6] World Health Organisation (WHO) (2000). Preventing Suicide: A Resource for Media Professionals.

[7] (2008). Media Guidelines for Reporting Suicide and Self-Harm.

[8] (2001). Reporting on Suicide: Recommendations for the Media.

[9] Fu K.W., Yip P.S.F. (2008). Changes in reporting of suicide news after the promotion of the WHO media recommendations. Suicide Life Threat. Behav..

[10] Niederkrotenthaler T., Sonneck G. (2007). Assessing the impact of media guidelines for reporting on suicides in Austria: Interrupted time series analysis. Aust. NZ J. Psychiatr..

[11] Pirkis J., Francis C., Blood R.W., Burgess P., Morley B., Stewart A., Putnis P. (2002). Reporting of suicide in the Australian media. Aust. NZ J. Psychiatr..

[12] Au J.S., Yip P.S., Chan C.L., Law Y.W. (2004). Newspaper reporting of suicide cases in Hong Kong. Crisis.

[13] Sisask M., Varnik A., Wasserman D. (2005). Internet comments on media reporting of two adolescents’ collective suicide attempt. Arch. Suicide Res..

[14] Hawton K., Williams K. (2001). The connection between media and suicidal behavior warrants serious attention. Crisis.

[15] Pirkis J., Dare A., Blood R.W., Rankin B., Williamson M., Burgess P., Jolley D. (2009). Changes in media reporting of suicide in Australia between 2000/01 and 2006/07. Crisis.

[16] Tatum P.T., Canetto S.S., Slater M.D. (2010). Suicide coverage in U.S. newspapers following the publication of the media guidelines. Suicide Life Threat. Behav..

[17] Michel K., Wyss K., Frey C., Valach L. (2000). An exercise in improving suicide reporting in print media. Crisis.

[18] Moher D., Liberati A., Tetzlaff J., Altman D.G. (2009). Preferred reporting items for systematic reviews and meta-analyses: The PRISMA statement. BMJ.

[19] Westerlund M., Schaller S., Schmidtke A., Wasserman D., Wasserman C. (2010). The Role of Mass-Media in Suicide Prevention. Oxford Textbook of Suicidology and Suicide Prevention: A Global Perspective.

[20] Motto J.A. (1967). Suicide and suggestibility: The role of the press. Am. J. Psychiatry.

[21] Motto J.A. (1970). Newspaper influence on suicide: A controlled study. Arch. Gen. Psychiatry.

[22] Phillips D.P. (1977). Motor vehicle fatalities increase just after publicized suicide stories. Science.

[23] Phillips D.P. (1979). Suicide, motor vehicle fatalities, and the mass media: Evidence toward a theory of suggestion. Am. J. Sociol..

[24] Ashton J.R., Donnan S. (1981). Suicide by burning as an epidemic phenomenon: An analysis of 82 deaths and inquests in England and Wales in 1978–9. Psychol. Med..

[25] Bollen K.A., Phillips D.P. (1982). Imitative suicides: A national study of the effects of television news stories. Am. Sociol. Rev..

[26] Stack S. (1983). The effect of the Jonestown suicides on American suicide rates. J. Soc. Psychol..

[27] Wasserman I.M. (1984). Imitation and suicide: A reexamination of the Werther effect. Am. Sociol. Rev..

[28] Kessler R.C., Downey G., Milavsky J.R., Stipp H. (1988). Clustering of teenage suicides after television news stories about suicides: A reconsideration. Am. J. Psychiatry.

[29] Stack S. (1988). Suicide: Media impacts in war and peace, 1910–1920. Suicide Life Threat. Behav..

[30] Kessler R.C., Downey G., Stipp H., Milavsky J.R. (1989). Network television news stories about suicide and short-term changes in total U.S. suicides. J. Nerv. Ment. Dis..

[31] Stack S. (1990). Audience receptiveness, the media, and aged suicide, 1968–1980. J. Aging Stud..

[32] Stack S. (1990). Divorce, suicide, and the mass media: An analysis of differential identification, 1948–1980. J. Marriage Fam..

[33] Stack S. (1990). A reanalysis of the impact of non celebrity suicides: A research note. Soc. Psychiatry Psychiatr. Epidemiol..

[34] Ishii K. (1991). Measuring mutual causation: Effects of suicide news on suicides in Japan. Soc. Sci. Res..

[35] Etzersdorfer E., Sonneck G., Nagel-Kuess S. (1992). Newspaper reports and suicide. N. Engl. J. Med..

[36] Stack S. (1992). The effect of the media on suicide: The Great Depression. Suicide Life Threat. Behav..

[37] Stack S. (1993). The media and suicide: A nonadditive model, 1968–1980. Suicide Life Threat. Behav..

[38] Sonneck G., Etzersdorfer E., Nagel-Kuess S. (1994). Imitative suicide on the Viennese subway. Soc. Sci. Med..

[39] Hassan R. (1995). Effects of newspaper stories on the incidence of suicide in Australia: A research note. Aust. NZ J. Psychiatry.

[40] Jobes D.A., Berman A.L., O’Carroll P.W., Eastgard S. (1996). The Kurt Cobain suicide crisis: Perspectives from research, public health and the news media. Suicide Life Threat. Behav..

[41] Stack S. (1996). The effect of the media on suicide: Evidence from Japan, 1955–1985. Suicide Life Threat. Behav..

[42] Etzersdorfer E., Sonneck G. (1998). Preventing suicide by influencing mass-media reporting. The Viennese experience 1980–1996. Arch. Suicide Res..

[43] Stack S. (2000). Media impacts on suicide: A quantitative review of 293 findings. Soc. Sci. Quart..

[44] Chung W.S., Leung C.M. (2001). Carbon monoxide poisoning as a new method of suicide in Hong Kong. Psychiatr. Serv..

[45] Etzersdorfer E., Voracek M., Sonneck G. (2001). A dose-response relationship of imitational suicides with newspaper distribution. Aust. NZ J. Psychiatry.

[46] Pirkis J., Blood R.W. (2001). Suicide and the media: Part I. Reportage in nonfictional media. Crisis.

[47] Stack S. (2002). Media coverage as a risk factor in suicide. Inj. Prev..

[48] Stack S. (2003). Media coverage as a risk factor in suicide. J. Epidemiol. Community Health.

[49] Etzersdorfer E., Voracek M., Sonneck G. (2004). A dose-response relationship between imitational suicides and newspaper distribution. Arch. Suicide Res..

[50] Hittner J.B. (2005). How robust is the Werther effect? A re-examination of the suggestion-imitation model of suicide. Mortality.

[51] Reisch T., Michel K. (2005). Securing a suicide hot spot: Effects of a safely net at the Bern Muenster Terrace. Suicide Life Threat. Behav..

[52] Shoval G., Zalsman G., Polakevitch J., Shtein N., Sommerfeld E., Berger E., Apter A. (2005). Effect of the broadcast of a television documentary about a teenager’s suicide in Israel on suicidal behavior and methods. Crisis.

[53] Stack S. (2005). Suicide in the media: A quantitative review of studies based on nonfictional stories. Suicide Life Threat. Behav..

[54] Sudak H.S., Sudak D.M. (2005). The media and suicide. Acad. Psychiatry.

[55] Tousignant M., Mishara B.L., Caillaud A., Fortin V., St-Laurent D. (2005). The impact of media coverage of the suicide of a well-known Quebec reporter: The case of Gaëtan Girouard. Soc. Sci. Med..

[56] Pirkis J.E., Burgess P.M., Francis C., Blood R.W., Jolley D.J. (2006). The relationship between media reporting of suicide and actual suicide in Australia. Soc. Sci. Med..

[57] Romer D., Jamieson P.E., Jamieson K.H. (2006). Are news reports of suicide contagious? A stringent test in six U.S. cities. J. Commun..

[58] Yip P.S.F., Fu K.W., Yang K.C.T., Ip B.Y.T., Chan C.L.W., Chen E.Y.H., Lee D.T.S., Law F.Y.W., Hawton K. (2006). The effects of a celebrity suicide on suicide rates in Hong Kong. J. Affect. Disord..

[59] Cheng A.T., Hawton K., Chen T.H., Yen A.M., Chang J.C., Chong M.Y., Liu C.Y., Lee Y., Teng P.R., Chen L.C. (2007). The influence of media reporting of a celebrity suicide on suicidal behavior in patients with a history of depressive disorder. J. Affect. Disord..

[60] Cheng A.T., Hawton K., Chen T.H., Yen A.M., Chen C.Y., Chen L.C., Teng P.R. (2007). The influence of media coverage of a celebrity suicide on subsequent suicide attempts. J. Clin. Psychiatry.

[61] Cheng A.T., Hawton K., Lee C.T., Chen T.H. (2007). The influence of media reporting of the suicide of a celebrity on suicide rates: A population-based study. Int. J. Epidemiol..

[62] Fu K.W., Yip P.S.F. (2007). Long-term impact of celebrity suicide on suicidal ideation: Results from a population-based study. J. Epidemiol. Community Health.

[63] Hagihara A., Tarumi K., Abe T. (2007). Media suicide-reports, Internet use and the occurrence of suicides between 1987 and 2005 in Japan. BMC Public Health.

[64] Fu K.W., Chan Y.Y., Yip P.S.F. (2009). Testing a theoretical model based on social cognitive theory for media influences on suicidal ideation: Results from a panel study. Media Psychol..

[65] Fu K.W., Yip P.S.F. (2009). Estimating the risk for suicide following the suicide deaths of 3 Asian entertainment celebrities: A meta-analytic approach. J. Clin. Psychiatry.

[66] Huh G.Y., Jo G.R., Kim K.H., Ahn Y.W., Lee S.Y. (2009). Imitative suicide by burning charcoal in the southeastern region of Korea: The influence of mass media reporting. Leg. Med. (Tokyo).

[67] Niederkrotenthaler T., Till B., Kapusta N.D., Voracek M., Dervic K., Sonneck G. (2009). Copycat effects after media reports on suicide: A population-based ecologic study. Soc. Sci. Med..

[68] Chen Y.Y., Liao S.F., Teng P.R., Tsai C.W., Fan H.F., Lee W.C., Cheng A.T. (2010). The impact of media reporting of the suicide of a singer on suicide rates in Taiwan. Soc. Psychiatry Psychiatr. Epidemiol..

[69] Chen Y.Y., Tsai P.C., Chen P.H., Fan C.C., Hung G.C., Cheng A.T. (2010). Effect of media reporting of the suicide of a singer in Taiwan: The case of Ivy Li. Soc. Psychiatry Psychiatr. Epidemiol..

[70] Chen Y.Y., Chen F., Yip P.S. (2011). The impact of media reporting of suicide on actual suicides in Taiwan, 2002–05. J. Epidemiol. Community Health.

[71] Kunrath S., Baumert J., Ladwig K.H. (2011). Increasing railway suicide acts after media coverage of a fatal railway accident? An ecological study of 747 suicidal acts. J. Epidemiol. Community Health.

[72] Niederkrotenthaler T., Voracek M., Herberth A., Till B., Strauss M., Etzersdorfer E., Eisenwort B., Sonneck G. (2010). Role of media reports in completed and prevented suicide: Werther v. Papageno effects. Br. J. Psychiatry.

[73] Queinec R., Beitz C., Contrand B., Jougla E., Leffondré K., Lagarde E., Encrenaz G. (2011). Copycat effect after celebrity suicides: Results from the French national death register. Psychol. Med..

[74] Stack S. (2000). Suicide: A 15-year review of the sociological literature part I: Cultural and economic factors. Suicide Life Threat. Behav..

[75] Phillips D.P. (1974). The influence of suggestion on suicide: Substantive and theroretical implications of the Werther effect. Am. Sociol. Rev..

[76] Kral M.J. (1994). Suicide as social logic. Suicide Life Threat. Behav..

[77] Stack S., Leenaars A.A. (1991). Social Correlates of Suicide by Age: Media Impacts. Life Span Perspectives of Suicide: Time-Lines in the Suicide Process.

[78] Pirkis J., Burgess P., Blood R.W., Francis C. (2007). The newsworthiness of suicide. Suicide Life Threat. Behav..

[79] Niederkrotenthaler T., Till B., Herberth A., Voracek M., Kapusta N.D., Etzersdorfer E., Strauss M., Sonneck G. (2009). The gap between suicide characteristics in the print media and in the population. Eur. J. Public Health.

[80] Blood R.W., Pirkis J. (2001). Suicide and the media. Part III: Theoretical issues. Crisis.

[81] Sisask M., Mark L., Värnik A. (2011). Internet comments elicited by media portrayal of a familicide-suicide case. Crisis.

[82] Stack S. (1987). Celebrities and Suicide: A taxonomy & analysis, 1948–1983. Am. Sociol. Rev..

[83] Stack S., Bowman B. (2011). Suicide movies: Social patterns, 1900–2009.

